# Supplementary material for: Serological evidence of West Nile virus circulation in Indonesia: Initial insights into an underrecognized flavivirus threat
Source: PLoS One. 2026 Feb 10;21(2):e0342511. doi: 10.1371/journal.pone.0342511 (PMC12890112; doi:10.1371/journal.pone.0342511)
Supplement: Attachment — Submitted filename: comment to author.docx [file pone.0342511.s002.docx]

**Comment to Author**

**Overall Comment:**

This is a well written and valuable study that provides updated serologic data on WNV exposure in a population often underrepresented in surveillance. The findings are timely and contribute to understanding possible WNV circulation in Indonesia. For future, inclusion of IgG testing and addition of negative serum tested for JEV sero-complexes could strengthen the assessment and make the study’s design and analysis more robust and informative.

Introduction

Line 60 – to be more precise and use commonly used term in current literature, acute flaccid myelitis

Line 61- to be more specific, the surveillance in humans only or including mosquitoes

Line 74- It was stated to investigate recent infection, but some of the samples were old sample from 3 years ago, the word recent infection is not suitable. It is understandable your focus in on non-febrile patient, therefore this sentence needs to be rephrased.

Line 75 – specify the general population location

Line 76- state the study focuses on non-febrile patient.

Methodology

Line 104 - state what type of ELISA. How the cut-off value of ISR is determined for both ELISA (WNV IgM and CDC MAC -ELISA)? The result is an OD or concentration of antibody?

Line 108 - where did you perform the CDC MAC-ELISA protocol?

State possible reason why IgG based ELISA and RT-PCR was not included in this study?

Add statistical analysis.

Discussion

While previous studies shown that WNV IgM can persist and sometimes indicate exposure, its duration is variable and less predictable in mild or asymptomatic infections. For more robust evidence of prior or cumulative exposure, including an IgG-based ELISA is recommended, ideally alongside IgM to distinguish recent versus past infection. Also how to confirm that the infection is recent if IgM-based ELISA could indicate both recent and exposure based on the study objective ( Line 74).

Have you considered screening serum samples from febrile patients that tested negative for dengue, Zika and JE? This could be a valuable approach for future studies.
