# Supplementary material for: Serological evidence of West Nile virus circulation in Indonesia: Initial insights into an underrecognized flavivirus threat
Source: PLoS One. 2026 Feb 10;21(2):e0342511. doi: 10.1371/journal.pone.0342511 (PMC12890112; doi:10.1371/journal.pone.0342511)
Supplement: Attachment — Submitted filename: Response to Reviewers_Rev1 05012026.docx [file pone.0342511.s004.docx]

02 January 2026

Manuscript: “Serological evidence of West Nile virus circulation in Indonesia: Initial insights into an underrecognized flavivirus threat**”**

Dear Editor,

We appreciate the insightful comments of the reviewers and have made appropriate revisions addressing all concerns. We believe that our manuscript has been greatly improved with the modifications that were suggested by the reviewers.

We look forward to hearing from you and would be glad to respond to any further questions and comments that you may have.

Best Regards,

Khin Saw Myint, MD, PhD

Reviewer #1: This paper provided evidence of West Nile Virus IgM positivity among people in Kalimantan and Papua. Unfortunately, there are some points that should be addressed to improve the paper quality.
*RESPONSE: We appreciate the reviewer’s constructive comments highlighting important issues and have revised accordingly, as detailed below.*

Comments
**Methods:**
Fourth paragraph, line 102 – 104: Add the reference for the reported sensitivity and specificity for the test.

*RESPONSE: A reference was added to reflect the revision.*

Fourth paragraph, line 108 – 109: Add more information on how to decide the final serostatus.

*RESPONSE: We appreciate the reviewer’s suggestion and have added a detailed explanation of how final serostatus was determined. These definitions are now included in the Methods section (Lines 121-126).*

**Results:**
Table 1: The numbers for N in Nunukan and Malinau sites were not matched with the numbers for sex and age group. Check again the numbers.

*RESPONSE: We thank the reviewer for spotting this typographical error. The values have been corrected.*

Table 2: The notes under the table should be added in the Methods section and in more detail.
*RESPONSE: It has now been integrated into the Methods section for clarity.*

**Discussion:**
Line 173 – 183: Are there any references for this paragraph?
*RESPONSE: Relevant reference has now been added to support this section.*

Last paragraph, line 191 – 193: “The modest sample size limits statistical power…” This study did not have “modest sample size”, it had “very small number of positive cases”. The authors did not mention anything about wanted to prove something by statistical test. Hence, it is quite confusing why suddenly the statistical power was mentioned as study limitation.

With two positive cases, it was no longer “reduces the ability to detect regional patterns”. The authors did not have the ability to detect regional patterns.

*RESPONSE: We thank the reviewer for this comment and agree that the original wording overstated our ability to perform statistical analyses. We have made the necessary correction (Lines 233-234).*

Reviewer #2: This is a well written and valuable study that provides updated serologic data on WNV exposure in a population often underrepresented in surveillance. The findings are timely and contribute to understanding possible WNV circulation in Indonesia. For future, inclusion of IgG testing and addition of negative serum tested for JEV sero-complexes could strengthen the assessment and make the study’s design and analysis more robust and informative.

*RESPONSE:* *We thank Reviewer 2 for the positive comments.*

**Introduction**
Line 60 – to be more precise and use commonly used term in current literature, acute flaccid myelitis

*RESPONSE: The term has been revised to acute flaccid myelitis.*

Line 61- to be more specific, the surveillance in humans only or including mosquitoes

*RESPONSE: The sentence has been revised to “The high frequency of inapparent infections in humans complicates surveillance efforts and contributes to substantial underreporting.”*

Line 74- It was stated to investigate recent infection, but some of the samples were old sample from 3 years ago, the word recent infection is not suitable. It is understandable your focus in on non-febrile patient, therefore this sentence needs to be rephrased.

*RESPONSE: We have revised the statement to “This study aimed to investigate the presence of WNV infections among non-febrile individuals.”*

Line 75 – specify the general population location

*RESPONSE: This has been added (Line 78). The detailed location of the general population sampled is described in the Methods section (Paragraph 1).*

Line 76- state the study focuses on non-febrile patient.

*RESPONSE: This has been clarified in the revised text.*

**Methodology**
Line 104 - state what type of ELISA. How the cut-off value of ISR is determined for both ELISA (WNV IgM and CDC MAC -ELISA)? The result is an OD or concentration of antibody?

*RESPONSE: We have revised the Methods section to specify the type of ELISA used and to explain how the cut-off values for both the InBios WNV IgM ELISA and the CDC MAC-ELISA were determined (Lines 107-129).*

Line 108 - where did you perform the CDC MAC-ELISA protocol?
State possible reason why IgG based ELISA and RT-PCR was not included in this study?
Add statistical analysis.
*RESPONSE: All laboratory assays, including the CDC MAC-ELISA, were performed at our laboratory at the Exeins Health Initiative (EHI), Indonesia.*

*IgG ELISA and RT-PCR were not included in this study due to the study’s focus on detecting IgM responses in non-febrile individuals rather than assessing long-term immunity (IgG) or active viremia (RT-PCR), combined with the limited diagnostic yield expected from the available specimens and budgetary constraints that precluded additional testing.*

*Statistical analysis has now been added in Lines 131-140.*

**Discussion**
While previous studies shown that WNV IgM can persist and sometimes indicate exposure, its duration is variable and less predictable in mild or asymptomatic infections. For more robust evidence of prior or cumulative exposure, including an IgG-based ELISA is recommended, ideally alongside IgM to distinguish recent versus past infection. Also how to confirm that the infection is recent if IgM-based ELISA could indicate both recent and exposure based on the study objective (Line 75-76).
*RESPONSE: We thank the reviewer for this important clarification. In the revised Discussion, we clarify that determining truly recent WNV infection was not possible in this study because only single serum samples were available (Lines 224-228).*

*We also agree that IgG-based ELISA, ideally interpreted together with IgM, would provide more robust evidence of cumulative or past exposure and help distinguish recent from historic infection. However, IgG testing was not included in the present study due to resource and logistical constraints, and we now explicitly acknowledge this as a limitation (Lines 230-233). We have revised the study objective and related text (Line 75) to avoid implying that IgM alone can confirm recent infection. The updated wording now states that the study aimed to investigate the presence* *of WNV infections among non-febrile individuals (Lines 75-76), rather than strictly “recent” infections.*

*Confirming recent infection would require additional diagnostic approaches such as demonstrating IgG seroconversion using paired acute and convalescent sera. These methods were beyond the scope of the current study and were not feasible given the available samples. This clarification ensures that the interpretation of IgM results remains accurate and does not overstate what IgM-based ELISA can determine.*

Have you considered screening serum samples from febrile patients that tested negative for dengue, Zika and JE? This could be a valuable approach for future studies.

*RESPONSE: This approach was not feasible for the present study, which focused exclusively on non-febrile individuals, and access to febrile samples fell outside the project scope. We have added this as a recommendation for future studies.*
